# The emergence of core eudicots: new floral evidence from the earliest Late Cretaceous

**DOI:** 10.1098/rspb.2016.1325

**Published:** 2016-12-28

**Authors:** Else Marie Friis, Kaj Raunsgaard Pedersen, Peter R. Crane

**Affiliations:** 1 Department of Palaeobiology, Swedish Museum of Natural History, Stockholm, Sweden; 2 Department of Geoscience, University of Aarhus, Aarhus, Denmark; 3 Yale School of Forestry and Environmental Studies, New Haven, CT, USA; 4 Oak Spring Garden Foundation, Upperville, VA, USA

**Keywords:** fossil flower, rosids, asterid, SRXTM, synchrotron X-ray microtomography, tricolporate pollen

## Abstract

Eudicots, the most diverse of the three major clades of living angiosperms, are first recognized in the latest Barremian–earliest Aptian. All Early Cretaceous forms appear to be related to species-poor lineages that diverged before the rise of core eudicots, which today comprise more than 70% of angiosperm species. Here, we report the discovery of a well-preserved flower, *Caliciflora mauldinensis*, from the earliest Late Cretaceous, with unequivocal core eudicot features, including five sepals, five petals and two whorls of stamens borne on the rim of a floral cup containing three free carpels. Pollen is tricolporate. Carpels mature into follicular fruitlets. This character combination suggests a phylogenetic position among rosids, but more specific assignment is precluded by complex patterns of character evolution among the very large number of potentially relevant extant taxa. The whorled floral organization is consistent with ideas that this stable pattern evolved early and was a prerequisite for more integrated patterns of floral architecture that evolved later. However, limited floral synorganization in *Caliciflora* and all earlier eudicot flowers recognized so far, calls into question hypotheses that substantial diversification of core eudicots had already occurred by the end of the Early Cretaceous.

## 1. Introduction

Hypotheses of relationships among living angiosperms recognize a species-poor basal grade, within which are embedded three major clades; eumagnoliids, monocots and eudicots. Soon after their first appearance in the fossil record about 135 Ma, Early Cretaceous angiosperms include diverse extinct taxa related to basal grade angiosperms (Austrobaileyales, Chloranthaceae and Nymphaeales), certain eumagnoliids (Laurales, Magnoliales and Piperales), early monocots (Alismatales) and basal grade eudicots (e.g. [1–4]). These Early Cretaceous assemblages contrast markedly with Late Cretaceous angiosperm assemblages that are dominated by fossils related to lineages of core eudicots. Core eudicots comprise more than 70% of living angiosperm species and both major clades within the group are well represented in the Late Cretaceous. Late Cretaceous rosids include a rich record of early Fagales (e.g. [5]) as well as diverse fossils related to other clades [1]. Late Cretaceous asterids include many taxa related to extant Cornales and Ericales (e.g. [1,6–9]), the two earliest diverging lineages of the group. The transition from Early Cretaceous floras, dominated by basal grade lineages of angiosperms, eumagnoliids and early eudicots, to Late Cretaceous floras dominated by core eudicots, occurred sometime between the mid-Albian and the Turonian–Santonian. Here, we describe a new flower from the earliest Late Cretaceous (earliest Cenomanian) of eastern North America with distinctive features of core eudicots. Together with an unnamed fossil flower of approximately similar age [10], this new discovery provides the earliest direct evidence of floral structure in early core eudicots and has implications for understanding the evolution of floral structure within this hyperdiverse clade of extant angiosperms.

## 2. Material and methods

The fossil material described here consists of one complete flower bud, one anthetic flower and three fragments of post-anthetic flowers, one of which has the remains of a stamen. All five specimens were recovered from samples of Potomac Group sediments (Mauldin Mountain samples 022, 116, 117) collected from the Elk Neck Beds at the Mauldin Mountain locality on the Elk Neck Peninsula, northeastern Maryland, USA (39°29'15″ N, 75°59'44″ W). The Elk Neck Beds of earliest Cenomanian age have also yielded inflorescences and flowers of *Mauldinia mirabilis* Drinnan, P. R. Crane, E. M. Friis & K. R. Pedersen [11], which is related to extant Lauraceae, fruits of *Couperites mauldinensis* K. R. Pedersen, P. R. Crane & E. M. Friis [12], which are of uncertain affinity among early diverging angiosperms, and pistillate and staminate flowers of *Spanomera mauldinensis* Drinnan, P. R. Crane, E. M. Friis & K. R. Pedersen [13], which are related to extant Buxales. For further details on the geology and age of the Mauldin Mountain assemblage, see [11].

The specimens are coalified and were treated following standard methods for Cretaceous mesofossils [1]. The flower bud was mounted on a brass stub for synchrotron radiation X-ray microtomography (SRXTM) at the Tomcat beamline of the Swiss Light Source, Paul Scherrer Institute, Switzerland [14]. It was measured using a ×20 objective with isotopic pixel size of 0.325 µm at 10 keV using a sCMOS detector and a 20 µm thick LAG:Ce scintillator screen and the specimen was vertically stacked (for more details on the technique, see [15]). Data derived from the SRXTM [16] were reconstructed and imaged using Avizo (v. 6.3, 7.1, 9.0.1, 9.1.1) software for computed tomography.

After SRXTM the flower bud was remounted on an aluminium stub for SEM scanning electron microscopy (SEM), sputter coated with gold, and examined using a Hitachi S-4300 Field Emission Scanning Electron Microscope at 2 kV. The four other specimens were also prepared for SEM in the same way.

## 3. Results

**Angiospermae**

**Core eudicots**

**Genus**. *Caliciflora* gen. nov.

**Type species designated here**. *Caliciflora mauldiniensis* sp. nov.

**Generic diagnosis**. Flower small, sessile, with an associated bract and two prophylls borne on a stout stalk. Staminate and pistillate organs in the same flower. Floral cup distinct. Perianth with five sepals and five petals borne on the rim of the floral cup. Sepals free, thick, with broad base and acute apex; sepal aestivation revolute-valvate. Petals free, broadening distally from a narrower base, keeled, with a thin lamina and median rib; petal aestivation open below, quincuncial above. Indumentum dense on the outer surfaces of the floral cup, sepals and petals composed of interlocking stellate hairs. Stamens in two whorls, minute with dithecate, tetrasporangiate and dorsifixed anthers. Pollen minute, tricolporate, psilate. Orbicules present. Gynoecium trimerous with three free carpels borne on the inside of the floral cup.

**Specific diagnosis**. As for the genus.

**Etymology**. Generic name from *calice* (Latin for cup) and *flos* (Latin for flower) and specific name from the Mauldin Mountain locality where the fossils were collected.

**Figure 1.**
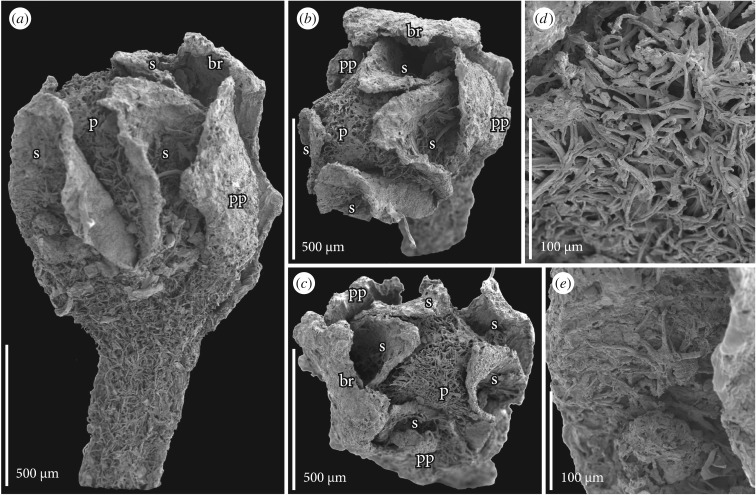
SEM images of flower bud of *Caliciflora mauldinensis* gen. et sp. nov. from the Late Cretaceous (earliest Cenomanian) Mauldin Mountain locality, MD, USA; holotype (PP53985; sample Mauldin Mountain 116). (*a*–*c*) Lateral (*a*) and apical (*b,c*) views of stalked flower bud showing subtending bract (br), two prophylls (pp), revolute-valvate sepals (s) and petals (p). (*d*) Detail of petal surface showing dense indumentum of interlocking stellate hairs. (*e*) Detail of sepal surface showing a stellate hair.

**Figure 2.**
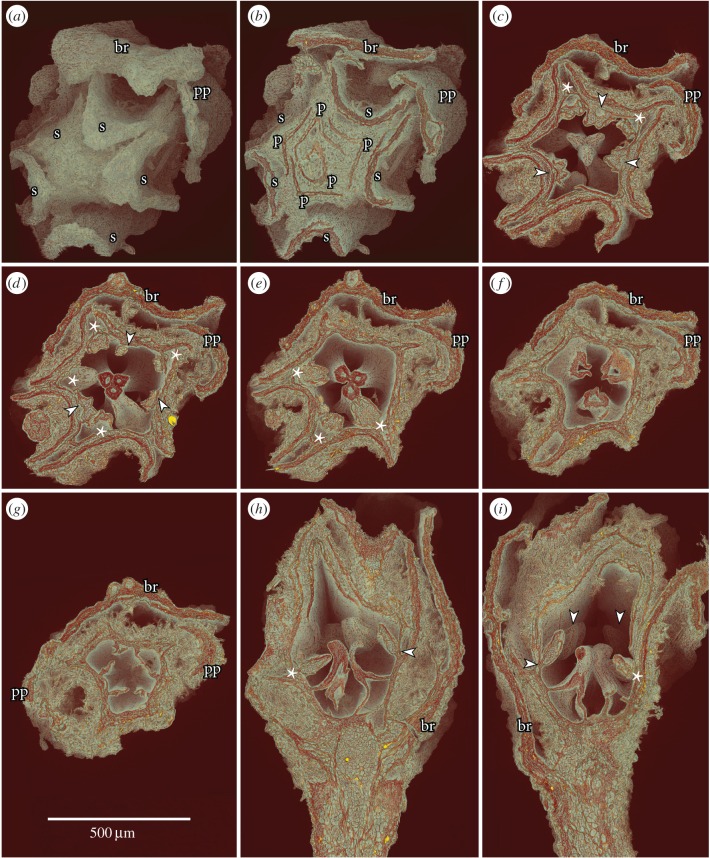
SRXTM volume renderings of flower bud of *Caliciflora mauldinensis* gen. et sp. nov. from the Late Cretaceous (earliest Cenomanian) Mauldin Mountain locality, MD, USA; holotype (PP53985; sample Mauldin Mountain 116). (*a*) Volume rendering of apical view of entire floral bud showing subtending bract (br), prophylls (pp) and five revolute-valvate sepals (s). (*b*–*g*) Transverse views of flower bud at different levels from apex (*b*) to middle of floral cup (*g*) showing subtending bract (br), two prophylls (pp), five revolute-valvate sepals (s), five petals (p), three antesepalous stamens (arrowheads), five antepetalous stamens (asterisks) and three carpels; note three closely appressed stigmas (*c*) and the free ventral margins of carpels below the stigmatic region (*f*,*g*) (*a*, not cut; *b*, section at level of orthoslice xy0766; *c*, section at level of orthoslice xy1756; *d*, section at level of orthoslice xy1916; *e*, section at level of orthoslice xy2044; *f*, section at level of orthoslice xy2233; *g*, section at level of orthoslice xy2427). (*h*,*i*) Longitudinal views of flower bud in median section showing floral cup surrounding the carpels and with antesepalous stamens (arrowheads) and antepetalous stamens (asterisks) on the rim of the floral cup (*h*, section at level of orthoslice xz1112; *i*, section at level of orthoslice yz0983).

**Figure 3.**
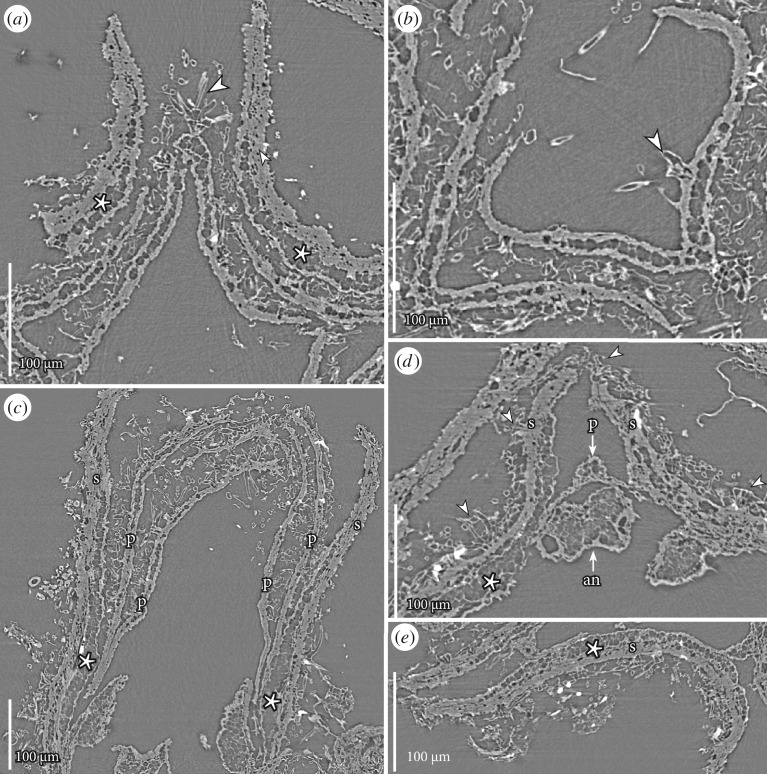
SRXTM reconstructions (orthoslices) of flower bud of *Caliciflora mauldinensis* gen. et sp. nov. flower from the Late Cretaceous (earliest Cenomanian) Mauldin Mountain locality, MD, USA; holotype (PP53985; sample Mauldin Mountain 116). (*a*) Detail of perianth in transverse section below the apex of the flower bud showing the thicker sepals with a layer of thin-walled hypodermal cells (asterisks), thin lamina of a folded petal with an almost glabrous inner surface and a dense indumentum on the outer surface; note the especially clear stellate hair (arrowhead) on the keel of the outer surface of the petal (orthoslice xy1200). (*b*) Detail of perianth in transverse section close to the apex of the floral bud showing folded petal with dense indumentum on the outer surface and scattered hairs on the inner surface (arrowhead) (orthoslice xy1050). (*c*) Longitudinal sections of sepals (s) and petals (p) showing dense indumentum; note sepals with dense, infilled cells towards the outside, and a layer of thin-walled hypodermal cells (asterisks) (orthoslice xz1485). (*d*) Detail of perianth and androecium in transverse section close to the rim of the floral cup showing sepals (s) with dense indumentum of stellate hairs on the outer surface and sepal margins (arrowhead), narrow base of petal (p, arrow) and an antepetalous anther (an, arrow) (orthoslice xy1900). (*e*) Detail of sepal (s) showing glabrous inner surface and dense indumentum of stellate hairs on the outer surface and sepal margins; note thin-walled cells on the inner surface of the sepal (asterisk) (orthoslice xy1950).

**Figure 4.**
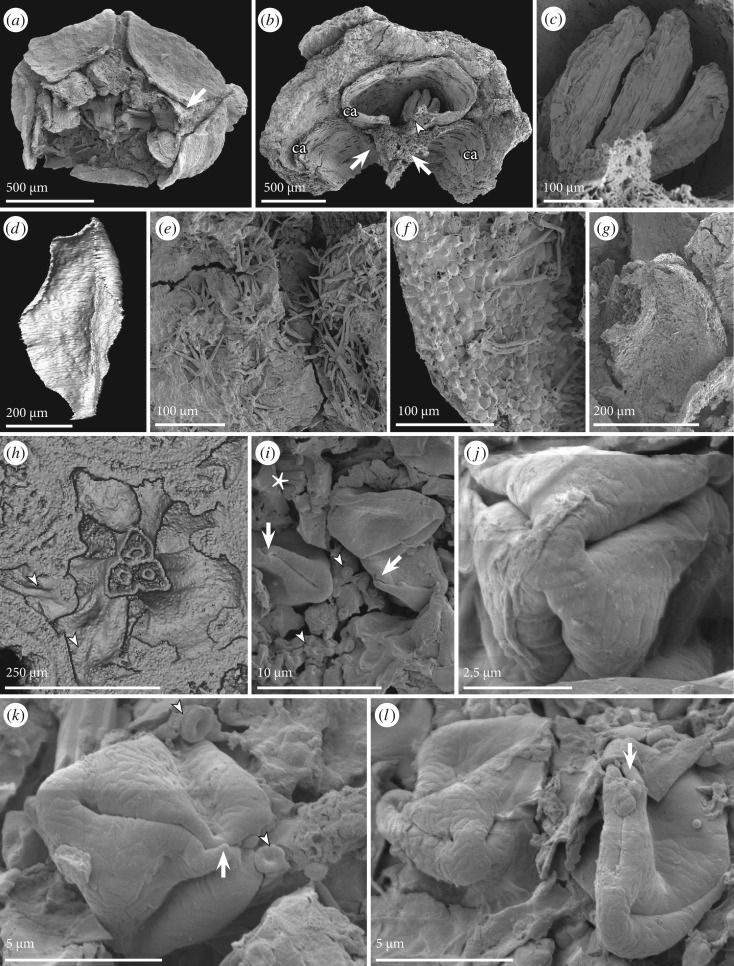
SEM (*a*–*c*, *e*–*g*, *i*–*l*) and SRXTM (*d, h*) images of carpels, ovules/seeds, stamens and pollen of *Caliciflora mauldinensis* gen. et sp. nov. from the Late Cretaceous (earliest Cenomanian) Mauldin Mountain locality, MD, USA. (*a*) Anthetic specimen in apical view showing five sepals, remnants of a keeled petal (arrow), six stamens and three carpels (PP34773, sample Mauldin Mountain 116). (*b*,*c*) Fragment of post-anthetic specimen in apical view (*b*) showing remains of three carpels (ca) and three elongated, anatropous, reticulate ovules/seeds (arrowhead) in the most complete carpel; note also remains of the slightly bulging margins of the two other carpels (arrows) (PP54159; sample Mauldin Mountain 117). (*d*) Surface rendering of adaxial surface of petal from specimen in figures 1–3 showing the gradual expansion of the thin petal lamina from the base and the distinct groove, which corresponds to the keel on the abaxial surface (PP53985; sample Mauldin Mountain 116). (*e,f*) Fragment of post-anthetic specimen showing the dense indumentum of stellate hairs on the surface of the sepals (*e*) and scattered on the papillate surface of the carpel (*f*) (PP54160; sample Mauldin Mountain 022). (*g*) Detail of the apical portion of an anther from anthetic flower in (*a*) showing numerous pollen grains *in situ* (PP34773, sample Mauldin Mountain 116). (*h*) Volume rendering of the floral bud cut at level of orthoslice xy1905 to show stamens with filament attached to dorsal side of the anthers (arrow heads) (PP53985; sample Mauldin Mountain 116). (*i*–*l*) Pollen grains are psilate to weakly regulate with three long colpi that reach almost to the pole; a narrow raised area (bridge) is seen at the middle of the colpi (arrows): note tiny, spherical orbicules (arrowheads) with a central depression adhering to the inner surface of the thecae and pollen grains (PP54161; sample Mauldin Mountain 022).

**Holotype designated here**. PP53985 (sample Mauldin Mountain 116); figure 1–4*d,h*.

**Paratypes**. PP34773 (sample Mauldin Mountain 116), PP54159 (sample Mauldin Mountain 117), PP54160, PP54161 (sample Mauldin Mountain 022).

**Type locality**. West of Mauldin Mountain, Elk Neck Peninsula, MD, USA, (39°29'15″ N, 75°59'44″ W).

**Type horizon and age**. Elk Neck Beds, Potomac Group; Late Cretaceous (Early Cenomanian; lowermost palynological Zone III).

**Description**. The taxon is based on a single flower bud (PP53985), one anthetic flower (PP34773) and three flower fragments preserved post-anthesis (PP54159–PP54161). The specimens are linked mainly by their identical stellate trichomes, similarities in the shape of the sepals, the presence of a floral cup and identical pollen grains *in situ* in specimens PP34773 and PP54161 and on the surface in PP54160. Sepals and petals are well-developed in the floral bud indicating that it was close to anthesis when fossilized.

The floral bud (figure 1–4*d,h*; electronic supplementary material, 1), the anthetic flower (figure 4*a*) and one of the post-anthetic flower fragments have both carpels and stamens. However, the bud is unusual in having carpels that appear partly open, with no signs of ovule initiation. It is therefore possible that the flower was functionally unisexual, and that only stamens developed to maturity. Alternatively, ovule development may have been interrupted by insect damage. There are burrows in the bud and an insect larva is present between the bract and sepals.

The flower and the associated bract and two prophylls are borne on a short, stout stalk (figures 1*a* and 2*h*,*i*). There is no other information about how the flower was borne on the plant. The flower bud is about 1.32 mm long and 0.75 mm in diameter, and has both staminate and pistillate organs preserved. There is also a distinct floral cup, about 0.17 mm deep and about 0.3 mm in diameter. The anthetic flower (figure 4*a*) is similar in size, about 1.2 mm in diameter, somewhat compressed and with fully formed staminate and pistillate organs.

The perianth is differentiated into an outer whorl of sepals and an inner whorl of petals. Sepal aestivation is revolute-valvate (figures 1*a–c*, 2*a*–*f* and 4*a*) and sepals have broad bases and acute apices. The post-anthetic flower fragments show that the sepals are persistent. On their ventral surface, the sepals have a conspicuous hypodermis of thin-walled, empty cells. This hypodermis is two to several cell layers thick in the median-basal region, but only one cell layer thick distally and along the sepal margins (figure 3*a*,*c–e*). Hypodermal cells on the dorsal surface of the sepals are distinctly different with amorphous contents that obscure the anatomical details of the sepal lamina, including the number of vascular bundles. The amorphous contents may indicate that these cells were mucilaginous or perhaps tanniferous (figure 3*a*,*c–e*).

Petals are present in the floral bud and in the specimen preserved at anthesis, but not in the post-anthetic specimens. Petals do not have a distinct claw, but broaden distally from a narrow base into a broad, keeled lamina with a prominent median rib and a single vascular bundle (figure 4*d*). Petals are folded longitudinally over the midrib and at least the inner petals appear conduplicate (figure 3*a*,*b*). The petals are narrow at the base, and widely separated by large interspaces. Petal aestivation is open at the base (figures 2*d* and 3*d*), but quincuncial above (figures 2*b*,*c* and 3*a*,*b*).

The androecium consists of two whorls of stamens. Anthers are almost sessile in the bud and filaments are also short in both of the more mature specimens (PP34773, PP54161). In the floral bud, the three stamens that are opposite the sepals (antesepalous) are borne on the rim of the floral cup. Five stamens opposite the petals (antepetalous) are borne slightly below this level (figure 2*c–e*,*h*,*i*). Anthers are tetrasporangiate, dithecal and dorsifixed (figure 4*a*,*h*). They are very small, up to about 0.15 mm long in the bud and about 0.4 mm long in the anthetic flower. Pollen grains are not obvious in the flower bud. Anthers in two of the more mature specimens have abundant minute pollen grains, about 10 µm long and 5 µm in equatorial diameter. Grains are prolate-rhomboidal in equatorial view (figure 4*i*,*k*,*l*) and semi-angular in polar view (figure 4*j*). Pollen grains have three long colpi, each with a small bridge in the middle of the colpi (figure 4*i*,*k*,*l*) indicating that the grains are tricolporate. The tectum is psilate-imperforate with weakly rugulate ornamentation. Tiny, rounded orbicules, about 1 µm in diameter, often with a small central depression, occur on the inside of the theca wall and the surface of the pollen grains (figure 4*i*,*k*), most probably indicating a secretory tapetum.

The three free carpels (figures 2*c–i* and 4*a*,*h*) are borne on the inside of the floral cup above the free space of the floral apex. In the flower bud, the carpels are fully open along the ventral suture below the stigmatic region (figure 2*c–g*). In this specimen, no ovules are developed and there is no indication of placentation. However, in the more mature specimens (figure 4*b*,*c*) placentation is ventral and apparently concentrated to the middle part of each carpel.

Ovules/seeds are exposed in one mature carpel (figure 4*b*,*c*). They are small, elongated, anatropous, with a reticulate outer surface, and do not fill out the locule. The fruitlets are dry follicles that open along their ventral sutures. The inner part of the fruit wall consists of transversely oriented fibrous sclereids. The outer epidermis of the fruit wall consists of equiaxial cells, each with a central papilla (figure 4*f*); no stomata have been observed on the carpel or fruit wall.

The outer surface of sepals and petals, as well as the outer surface of the floral cup is covered by a dense indumentum of interlocking stellate hairs each composed of up to about 10 long unicellular elements that radiate from a central point (figures 1*a–e*, 2*a–i*, 3*a–e* and 4*e*). Scattered stellate hairs also occur along the margins of the sepals (figure 3*d*) and on the inner surface of the petals in the apical region (figures 2*i* and 3*b*,*c*). The inner surface of the sepals and the median and basal parts of the petals are glabrous (figure 3*a*,*c*–*e*).

## 4. Discussion

### (a) Systematic assessment

The combination of characters in *Caliciflora*, including the actinomorphic organization with an open floral cup, pentamerous whorls of free sepals and petals, two whorls of free stamens, tricolporate and tectate-imperforate pollen, one whorl of three free carpels and follicular fruitlets, unequivocally place the fossil taxon among core eudicots. In addition, while flowers with a floral cup, together with free petals (choripetalous) and an apocarpous gynoecium, are found in several groups of rosids, to our knowledge this combination of features is not recorded among asterids or in any other group of core eudicots.

Resolving the phylogenetic position of *Caliciflora* among extant rosids is complicated by the vast number of living species (around 70 000 extant species, [17]), many of which have not been studied in detail, combined with the absence of clear morphological synapomorphies that correspond to the higher level groupings, orders and families recognized based largely on molecular data (e.g. [17–24]). A further problem is that floral morphology among rosids exhibits considerable variation, and sometimes there are strong similarities in unusual features between taxa that are widely separated phylogenetically (e.g. flowers of *Anisophyllea* (Anisophylleaceae, Cucurbitales: fabids) and *Ceratopetalum* (Cunoniaceae, Oxalidales: COM)) [25]. These difficulties, coupled with uncertainties over deep relationships in the rosids, complicate characterization of major clades using flower morphology [22,26–28] and there are no recent comprehensive attempts to evaluate the evolution of floral characters in the group as a whole. We therefore compare *Caliciflora* to extant rosids only in general terms based on the extensive literature, as well as the few discussions of floral structure that have taken into account new molecular models of angiosperm relationships (e.g. [25,26,28–41]).

One of the most distinctive characters of *Caliciflora* is the floral cup, which is also characteristic of many rosids, where it occurs scattered in three major clades (N-fixing clade, COM clade and malvids), as well as in Myrtales [28,42–45] and Crossomostomatales [33]. In the N-fixing clade, a floral cup is present in certain Rosales [40,41] and Cucurbitales [31], while in the COM clade, it occurs in Celastrales [32], Oxalidales [30] and Malpighiales [29,35–37,46]. Among malvids, a floral cup occurs in certain Brassicales [47], Huerteales [28] and Sapindales [48,49]. Actinomorphic flowers with free perianth parts are also characteristic of many rosids, and typically such flowers are pentamerous with a well-differentiated calyx and corolla as in *Caliciflora*. The revolute-valvate sepal aestivation of *Caliciflora* is also a feature of certain rosids and occurs scattered in the Cucurbitales (Anisophylleaceae, [31]), Malpighiales (Rhizophoraceae and Erythroxylaceae, [36]) and also in Oxalidales (Cunoniaceae and Tremandraceae, [30]).

A characteristic feature of many rosids is flowers with the same number of floral organs in all whorls (isomerous) including the gynoecial whorl. However, monocarpellate, bicarpellate and tricarpellate forms often occur in typically isomerous groups, and some groups of rosids, for example, many Malpighiales [46] are characterized by heteromerous flowers with one to three carpels. Also common in several groups of rosids is apocarpy and in the N-fixing clade it occurs in certain Rosaceae, as well as Surianaceae and Quillajaceae (both fabids). In Rosaceae, dry, follicular fruitlets with several seeds borne centrally on ventral placentae, as well as open ventral sutures, are known in some members of subfamily Amygdaloideae [50].

The androecial features of *Caliciflora* are less informative systematically, but are also consistent with the androecial structure of some members of the N-fixing clade. For example, while most flowers of Rosaceae have 15 or more stamens, flowers with fewer stamens occur in the North American cushion plant, *Kelseya uniflora* (S. Wats.) Rydb. [51]. Reduction of stamen number in one of the stamen whorls, as seen in the *Caliciflora* flower bud, occurs also in Surianaceae (Fabales).

Pollen morphology among rosids is diverse, but small, tricolporate grains similar to those of *Caliciflora* are common in many taxa. Often the tectum is reticulate, microreticulate or foveolate, but grains with a finely rugulate tectum as in *Caliciflora* occur in many taxa in the N-fixing clade, including in Fabaceae (e.g. [52–55]), Surianaceae and Quillajaceae [56], and Rosaceae (e.g. [57,58]).

The stellate hairs of *Caliciflora* are distinctive. Similar hairs occur on the outer surface of sepals and petals in Rhizophoraceae, and in the closely related Ctenolophonaceae [36], as well as on the outer surface of the sepals in some Cunoniaceae and Tremandraceae [30,59]. In flower buds of Rhizophoraceae and Ctenolophonaceae, the perianth parts are congenitally connected by the hairs in the overlapping regions [36], which also appears to be the case for *Caliciflora*. Congenital connection of sepals by hairs is also reported for Cunoniaceae [30]. Stellate hairs similar to those of *Caliciflora* are not widespread in Rosaceae, but occur in a few taxa (e.g. *Sorbaria*) [60].

### (b) Implications for the origin of core eudicots

**Figure 5.**
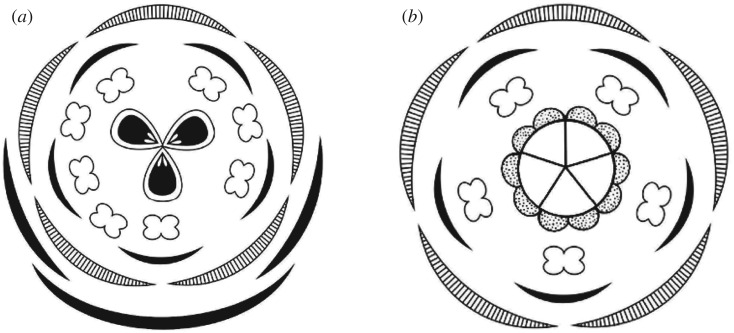
Floral diagrams of *Caliciflora mauldinensis* gen. et sp. nov. from the Late Cretaceous (earliest Cenomanian) Mauldin Mountain locality, MD, USA (*a*) and the Rose Creek Flower [10] from the latest Early Cretaceous or earliest Late Cretaceous (latest Albian–earliest Cenomanian) Rose Creek locality, NE, USA (*b*).

*Caliciflora mauldinensis* from the earliest Cenomanian, very close to the Early–Late Cretaceous boundary, establishes a minimum age of about 100 Myr for the origin of core eudicots. Setting aside the report of a rosid flower from Burmese amber [61], the age of which is uncertain (possibly Cenomanian), the only other mid-Cretaceous core eudicot flower currently known from around the Early–Late Cretaceous boundary is the Rose Creek flower reported from the Dakota Formation [10], which is of broadly similar age, or perhaps very slightly older [62]. While *Caliciflora* and the Rose Creek flower differ dramatically in size, their floral architecture is fundamentally similar. Both are regular actinomorphic and bisexual flowers with whorled, pentamerous organization and free floral parts (figure 5*a*,*b*), a prerequisite for the development of more integrated patterns of floral architecture [63] that accompanied the rapid diversification of core eudicots through the Late Cretaceous and Cenozoic [1,64].

Subsequent to the earliest Cenomanian there are only few fossil floras of Late Cenomanian or Turonian age. Currently no core eudicot flowers have been described from the Late Cenomanian flora of the Bohemian Basin [65–67] or from the Cenomanian–Turonian Sarbay flora [68,69]. The mesofossil flora from Old Crossman Clay Pit of possible Turonian age (e.g. [8,70]) does contain abundant flowers of core eudicots. However, the Old Crossman flora is very similar in many respects to younger mesofossil floras from the Santonian–Campanian of North America [1]. The interval between the first appearance of early core eudicot flowers (*Caliciflora,* Rose Creek flower) and later flowers with more pronounced synorganization (e.g. *Raritaniflora tomentosa* Crepet, Nixon & Daghlian) is therefore a minimum of about 10, or possibly as much 15–20 Myr.

The Late Cretaceous record of both mesofossils and palynofloras both suggest an extensive post-Cenomanian radiation of core eudicot angiosperms [1,71,72]. However, at the level of *Caliciflora* and the Rose Creek flower, and also in older sediments, all other angiosperms that are known so far appear to be related to early diverging lineages of angiosperms [1], including early diverging lineages of eudicots (taxa related to Lardizabalaceae and other members of Ranunculales, Buxales, Platanaceae and Nelumbonaceae [1,3,73]). Similarly, among the 21 species of fossil leaves reported from the Rose Creek locality, 70% of the leaf species and 90% of the leaf specimens appear to be related to magnoliids or magnoliid grade angiosperms [74]. Four species were unassigned. Only two species were assigned to core eudicots [74].

## 5. Conclusion

The characters of *Caliciflora* unequivocally place this new fossil angiosperm flower among core eudicots, most likely among the rosids rather than asterids, perhaps near the base of the N-fixing clade. Flowers with a floral cup, heteromerous organization, tricolporate, rugulate-tectate pollen and an apocarpous gynoecium are common at this level of angiosperm evolution, including in certain Rosaceae, and also in the fabalean family Surianaceae. More precise systematic placement is precluded by incomplete knowledge of floral structure over a very large number of extant species as well as problems of defining the currently recognized rosid lineages based on floral features alone. *Caliciflora* and the Rose Creek flower establish a minimum age for core eudicots that is broadly in agreement with ideas based on molecular data that suggest a major radiation of core eudicots in the mid-Cretaceous [17,75–77]. However, hypotheses suggesting that a significant diversification of core eudicots was already underway in the Early Cretaceous [78] are more problematic. Direct fossil evidence of core eudicots prior to around the Early Cretaceous–Late Cretaceous is currently lacking.

## Supplementary Material

ESM1

## Data accessibility

The specimens are housed in the palaeobotanical collections of the Field Museum of Natural History, Chicago, USA (PP). Raw data from the SRXTM study are stored at the Swedish Museum of Natural History, Stockholm, Sweden (raw data PP53985a_B1_B2_).
