# Supplementary material for: The emergence of core eudicots: new floral evidence from the earliest Late Cretaceous
Source: Proc Biol Sci. 2016 Dec 28;283(1845):20161325. doi: 10.1098/rspb.2016.1325 (PMC5204160; doi:10.1098/rspb.2016.1325)
Supplement: ESM1 [file rspb20161325supp1.docx]

**Electronic supplementary material**

***Proceedings of The Royal Society B: Biological Sciences***

The emergence of core eudicots: new floral evidence from the earliest Late Cretaceous

Else Marie Friis, Kaj Raunsgaard Pedersen, Peter R. Crane

Included is one movie clip (.mpg) containing reconstructed sections through a volume rendering of specimen PP53985. The movie clip was created based on SRXTM data using Avizo (version 9.1.1) software.
